# Event and Comment

**Published:** 1919-09

**Authors:** 


### DENTAL REGISTER

THE MAGAZINE OF DENTISTRY

Vol. LXXIII SEPTEMBER, 1919 No. 9

### EVENT AND COMMENT

**Trouble at Vanderbilt**

The chancellor of Vanderbilt University announced at the close of the last session of the dental department that it would not reopen unless financial aid to the extent of a half million dollars endowment could be secured. The reason for this seems to be that the income is insufficient to pay the increased cost of maintainance. The fact that the increase in standards requires more teachers who will give the major part of their time to instruction has practically doubled the cost for this item of instruction alone. The introduction of new courses requires a material increase in equipment as well as many other expensive additions in the way of buildings and rearrangement of rooming facilities. Then the increase in the time required of students for completing the course has made a decided decrease in the number of students applying for the course, which will of course make an appreciable reduction in the usual income.

It is very easy to see that the dental educational problem is a serious one for all schools that are not financially well supported, and we are not surprised that even so good a school, and one that can not well be spared, has taken so radical a step as to discontinue its work. We would not be surprised if others will not be forced to do likewise in the next year. The fact that Dr. Henry W. Morgan, the Dean, has broken down in health and has been compelled to give up his practice for a long period of complete rest probably has had much to do with the decision to close the school as he has devoted the better part of his time for many years to the executive and teaching duties and carried the larger part of the burden. No one will ever know, except possibly his intimate associates, how much of care and expense his work in the effort to maintain his school on the same ideal basis of professionalism that he established in his private practice has cost him. Dr. Morgan contends and always has contended for the highest ideals in the conduct of dental colleges, and no man in the dental profession is more honored and respected for his personal integrity and professional ideals and attainments. The dental educational problem in the South has always been a difficult one to harmonize with that of the Northern States, and we all know that Dr. Morgan has had a most difficult task to conduct a school that would be consistent with his professional ideals and be practicable under the conditions with which he had to contend.

We understand the dentists of Nashville and other alumni of the school are ready to guarantee any deficit in the expense of conducting the school for the next four years, and there is a strong probability that its doors will again open in October. We sincerely hope this may be the case.

**A New Type of Dental Office**

When the American Red Cross representatives arrived in Servia, they found only one dentist on the entire front. The frightful health conditions necessitated prompt action, but the American Red Cross was used to that and speedily brought overseas American dentists and dental supplies.

Unfortunately their offices could not be stationary as the railways had been destroyed by the Austrians, so dental chairs were screwed on trucks, and the dentists and all equipment necessary for a modern dental emporium, traveled over the Servian front, ending the miseries of both soldier and civilian.

Dental clinics were also established in France and Belgium and here the greatest miracle of dentistry resulted. The children became fond of the dentist. They would eagerly await their turn to get in the chairs and the coming of the dentist would be acclaimed with shouts of delight.

